# Threshold-varying integrate-and-fire model reproduces distributions of spontaneous blink intervals

**DOI:** 10.1371/journal.pone.0206528

**Published:** 2018-10-30

**Authors:** Ryota Nomura, Ying-Zong Liang, Kenji Morita, Kantaro Fujiwara, Tohru Ikeguchi

**Affiliations:** 1 Graduate School of Engineering, Tokyo University of Science, Tokyo, Japan; 2 Graduate School of Engineering, The University of Tokyo, Tokyo, Japan; 3 Graduate School of Education, The University of Tokyo, Tokyo, Japan; 4 International Research Center for Neurointelligence, The University of Tokyo, Tokyo, Japan; 5 Faculty of Engineering, Tokyo University of Science, Tokyo, Japan; University of Muenster, GERMANY

## Abstract

Spontaneous blinking is one of the most frequent human behaviours. While attentionally guided blinking may benefit human survival, the function of spontaneous frequent blinking in cognitive processes is poorly understood. To model human spontaneous blinking, we proposed a leaky integrate-and-fire model with a variable threshold which is assumed to represent physiological fluctuations during cognitive tasks. The proposed model is capable of reproducing bimodal, normal, and widespread peak-less distributions of inter-blink intervals as well as the more common popular positively skewed distributions. For bimodal distributions, the temporal positions of the two peaks depend on the baseline and the amplitude of the fluctuating threshold function. Parameters that reproduce experimentally derived bimodal distributions suggest that relatively slow oscillations (0.11–0.25 Hz) govern blink elicitations. The results also suggest that changes in blink rates would reflect fluctuations of threshold regulated by human internal states.

## Introduction

Spontaneous blinking is the most frequent eye-closing behaviour in daily life [1]. Humans spontaneously blink 20–30 times per minute [2]. This is approximately 5–10 times as many as the necessary frequency to maintain the humidity of eye surfaces [3].

In recent years, it has been hypothesized that such frequent blinking could play an important role in adaptive human behaviours [4], [5]. Participants in a laboratory experiment tended to blink immediately after the emergence of intermittently presented visual stimuli [6] indicating that people reliably receive visual information avoiding oversight errors. Similarly, researchers have reported that viewers were likely to blink at implicit breaks in expert storytelling performances [7]. These findings suggest that people know when to blink through interaction with external inputs. As a result, temporal shifts of attention are guided by professional performances, with an emerging synchronizations of blinking. Neurological research further showed that spontaneous blinks contribute to disengaging attention from audio-visual stimuli [8]. Owing to this function, people would be able to allocate attention to new targets immediately after blinking. Thus blinking could be a means for humans to efficiently gather information from the huge amount of surrounding audio-visual stimuli.

Although numerous experimental studies have been developed, little theoretical research using mathematical models has been carried out. The one-dimensional stochastic diffusion (OSD) model has been proposed as a mathematical model of spontaneous blinking [9]. This model assumes a blink generator in which electrical potential varies depending on the external inputs of corneal stimulation such as dryness, dust, or muscle fatigue. The electrical potential varies as Brownian motion process, resulting in a blink when the potential reaches a threshold. The potential exponentially decays to a constant value when the blink generator receives no inputs. Thus, intervals between spontaneous blinks are formulated as a first-passage-time to a constant threshold. According to [9], burst patterns in blinking can be explained by assuming that the threshold was shifted lower when the participants were drowsy.

Human blinking rates, however, vary in a few tens of seconds while watching an audio-visual stimulus [10]. A realistic model should account for this variation. In addition to such temporal characteristics, changes in blinking rates often provide less common distributions of inter-blink intervals (IBIs) in cognitive tasks [6], [11]. Thus, an adequate model should reproduce the diverse distributions of spontaneous blinking. The OSD model cannot reproduce distributions of IBIs because of its stochastic nature and constant threshold.

In this paper, we propose a leaky integrate-and-fire (LIF) model with a variable threshold to represent the fluctuation of internal states of human blinks. First, we examine the reproducibility of the distributions of IBIs by the OSD model, however, the OSD cannot reproduce experimental results. Then, we show that the proposed LIF model reproduces a variety of distributions such as the positively skewed, normal, peak-less, and bimodal distributions of IBIs. Finally, we explore the parameters that reproduce the distributions of IBI reported in a classical experimental study.

## Model of human spontaneous blinks

### One-dimensional stochastic diffusion model

In this model, changes in the potential *X* of the blink generator are governed by the following equation:

$$\begin{array}{c} dX\left(t\right)=(-\frac{X(t)}{\beta }+\mu )dt+\phi dW\left(t\right), \end{array}$$ (1)

with an initial condition *X*(0) = *X*_0_.

In Eq (1), *W* is a Wiener process that is characterized by spontaneous decay *β* (> 0), average input *μ* (−∞ < *μ* < ∞), and a noise term of *ϕ* (> 0) for a random process. This stochastic differential equation is formally equivalent to the Ornstein-Uhlenbeck process. The interval between one blink and the next (IBI) can be expressed as a first-passage-time density function, which is defined by the time duration between the initial potential *X*_0_ and the time to pass the threshold potential.

The OSD model is based on the Ornstein-Uhlenbeck process and therefore the potential *X* obeys the mean reversion law [9]. If we took *P*(*ω*|*α*, *t*) as the probability that a stochastic variable *α* is given when *t* = 0 whereby we gain *ω* at time *t*, in this model, *P*(−∞|*X*_0_, *t*) = 0. According to Hoshino [9], this mathematical assumption represents the physiological nature of a blinking generator that reliably repeats to active blinking within a finite time period without assuming a reflecting boundary.

The results of numerical simulations demonstrated that the OSD model can reproduce the positively skewed distribution of experimentally observed IBI [9]. However, this model does not reproduce the other previously reported distributions of IBI (See, Fig B in S1 File).

### Leaky integrate-and-fire model with a variable threshold

Although the primary physiological function of blinking is to prevent dryness of eye-surfaces, cognitive functions of human blinks have also been reported [7], [12]. A human blinks in accordance with semantic segmentations of audio-visual information. For example, people tend to blink after looking at punctuation marks in reading tasks [12] and immediately after listening to the punch line of jokes while viewing a storytelling performance [7]. Neurological research indicated that spontaneous blinks contribute to disengaging attention from audio-visual stimuli [8]. Cognitive load is integrated while audio-visual information is continuously accumulated. When people blinks, however, the cognitive load is reset by attentional disengagement where a part of audio-visual information is transmitted to the next processing stage. These facts indicate that we can model the biophysical changes in an internal value of a blink generator which is driven by cognitive load as well as by physiological inputs such as dryness and fatigue of muscle.

As one of the possible models, we used a leaky integrate-and-fire model with a variable threshold to represent such a blink-and-reset mechanism. The leaky integrate-and-fire models have been used as models of changes in membrane potential of a single neuron [13]. Human blinking is a macroscopic phenomenon that involves several brain areas. However, as far as we could assume that integrate-and-reset mechanism as a plausible postulation, the leaky integrate-and-fire model is suitable for human blinking as well.

As a possible mechanism for blinking intervals providing a variety of distributions, we assumed that the changes in blink rates are regulated by internal states that could vary in accordance with external stimuli. To construct the model, we assume a simply formulated situation where a background oscillation exists as a regulator of frequent human blinking. Such oscillation would emerge spontaneously as a result of physiological rhythms in addition to the rhythm induced by the external stimuli during an experimental task that requires visual attention. In this study, we consider a leaky integrate-and-fire model with a variable threshold [14].

The potential *V* of blinking generator is governed by

$$\begin{array}{c} \frac{dV}{dt}=-cV+I+\xi , \end{array}$$ (2)

where *c* is a constant decay term and *I* is an external input with intensity *b*. The last term represents the Gaussian noise *ξ* ∼ *N*(0, *σ*^2^) derived from the random fluctuation of external stimuli. The noise *ξ* = 0 when *σ* = 0.

One way to extract a particular rhythmic process in a physiological system is to set a variable threshold function [15]. Then, we introduced the following threshold function *θ*(*t*) determined by

$$\begin{array}{c} \theta \left(t\right)=a+k\sin\frac{2\pi t}{\tau }, \end{array}$$ (3)

where *a* is the baseline constant, *k* is the amplitude coefficient, and *τ* is the period. When *V* reaches the threshold, it immediately elicits a blink.

**Fig 1:**
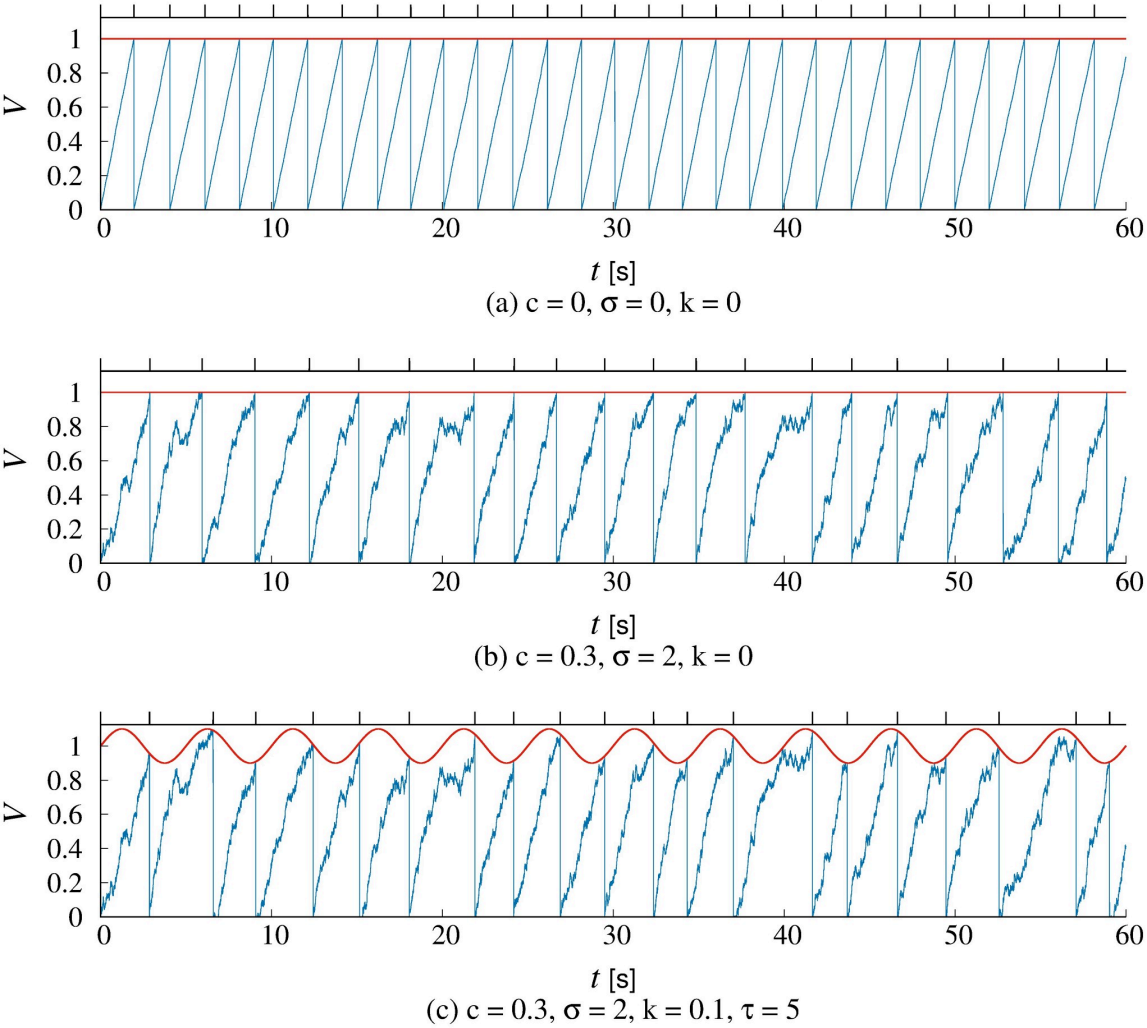
Results by the LIF model with (a), (b) a constant and (c) a variable threshold. The *V* increases with integrating the binomial input *I*. The parameter *c* is the decay term and the parameter *σ* is the standard deviation of noise *ξ*. The baseline of the threshold function *a* = 1. (a) There are no decay and no noise, i.e., *c* = 0 and *σ* = 0. (b) There is no noise, i.e., *σ* = 0. (c) The threshold is time-varing with the amplitude *k* and the period *τ* where the decay and the noise exist.

Fig 1(a) and 1(b) show the typical pattern when *a* = 1 and *k* = 0, i.e. *θ*(*t*) = 1. In a simple case of a perfect integrator without decay and noise, i.e. *c* = 0 and *σ* = 0, *V* demonstrates a monotone increasing with accumulating non-negative external inputs *I* (Fig 1(a)). Even when the threshold is constant, *V*, in the integrate-and-fire model, behaves in a complex way due to the decay term *c* and the noise *σ* = 0, resulting in the creation of irregular IBIs (Fig 1(b)). The parameter *k* determines the amplitude of the threshold function *θ*(*t*). Owing to the nonlinearity of the varying threshold function *θ*(*t*), IBIs can show rather complex patterns even if the external input *I* is constant. The model was deposited in BioModels Database [16] and assigned the identifier MODEL1810190001.

Previous researches have revealed the effect in a modulation of the current in LIF models of a neuron numerically and analytically [13], [17], [18]. A modulation of the current can be mathematically transformed to the variations of threshold. Therefore, the LIF model with a variable threshold would provide results that correspond to the previous research on a neuron. However, the LIF model would also be useful to understand statistical behavior of the human blinkings if the LIF model fit the data from physiological experiments.

## Numerical simulation and analysis

### Parameters

To the best of the authors’ knowledge, no mathematical proof provides that first-passage-time density functions of the Ornstein-Uhlenbeck process always exhibits positively skewed distributions. Thus, the ODS model [9] may reproduce a variety of distributions when specific parameters are set. Hence, we re-examined the distributions simulated by the OSD model. In this replication, threshold potential was set to 1.0 and the parameters of the Ornstein-Uhlenbeck process were set as shown in Table 1 to cover the typical ranges of decay *β* and input *μ* that elicit blinking at realistic intervals. In the numerical experiments, the parameters *β*, *μ*, and *ϕ* are increased by the values denoted in the third column of Table 1.

**Table 1:** Parameters used in the OSD model.

	range	an increment
*β*	[0.01, 10.0]	0.01
*μ*	[0.1, 10.0]	0.1
*ϕ*	[0.5, 1.0]	0.05

In all simulations, the time step was set to *dt* = 0.001 s. The total time for observation was 50 min (= 3, 000 s) to gain enough occurrences of IBI to estimate the distribution of human spontaneous blinking [3].

On the other hand, in the simulations of the proposed model, parameters were set as follows: the intensity of the external input *I* of which intervals obey a binomial distribution was set to *b* = 1. To explore a relatively wide range of intensities for the inputs, a constant threshold baseline *a* = 1 was set. When we assume the simple case with *c* = 0 and *σ* = 0, it is necessary to accumulate non-negative inputs 1, 000 times because *b* × *dt* = 0.001. Taking into account the binomial distribution of *I*, 2, 000 steps were needed on average to reach the threshold baseline. In other words, the variable *V* reaches the threshold in an average of 2 s. For instance, in case that *k* = 0.20, this corresponds to a maximum deviation 1/5 from the threshold baseline when *a* = 1. In case that *k* = 0.0, however, the threshold is a constant *θ*(*t*) = *a* because

$$\begin{array}{c} k\sin\frac{2\pi t}{\tau }=0. \end{array}$$ (4)

The period *τ* corresponds to the frequency of the threshold function *θ*(*t*). For example, the frequency of the threshold is 0.1 Hz for *τ* = 10 s and 10.0 Hz for *τ* = 0.1 s. Fig 1(c) shows the typical pattern when *a* = 1, *k* = 1/10, and *τ* = 5 s, i.e.

$$\begin{array}{c} \theta \left(t\right)=1+\frac{1}{10}\sin\frac{2\pi t}{5}. \end{array}$$ (5)

### Evaluation of distribution

Based on observation of human blinking behaviours, Ponder and Kennedy [19] reported four types of distributions of IBI. Although this study is classical, we focused on this study because it had reported all of known distributions. Moreover, the distributions were obtained from sufficient number of participants with using a certain procedure. Variations of distributions were consistent with that obtained in the following experimental studies [2], [5]. Thus, Ponder & Kennedy’s [19] four types of distributions of IBI are very informative even in recent years. According to [19], the results show that most common distribution was positively skewed (62.0%, 31/50 people). The authors also observed peak-less distributions (22.0%, 11/50), bimodal distributions (12.0%, 6/50), and normal distributions (4.0%, 2/50).

We evaluated the peaks of simulated distributions of IBIs using kernel estimation of probability density. The kernel density function ${\hat{f}}_{h}(x)$ was estimated as

$$\begin{array}{c} {\hat{f}}_{h}\left(x\right)=\frac{1}{nh}\sum_{i=1}^{n}K\left(u\right). \end{array}$$ (6)

We used a Gaussian kernel function, which is described as

$$\begin{array}{c} K\left(u\right)=\frac{1}{\sqrt{2\pi }}e^{-u^{2}/2}, \end{array}$$ (7)

where

$$\begin{array}{c} u=\frac{x-x_{i}}{h}. \end{array}$$ (8)

In this equation, *x*_*i*_ was the *i*th observed value and *h* was the bandwidth, *n* was the total number of *x*_*i*_. For kernel density estimations, we used the C++ library [20] in which the optimal bandwidths *h* were calculated as the integral over the square of the curvature using the trapezoidal rule.

We then estimated the number of peaks in the simulated distributions by applying the peak-finding algorithm [21]. In order to detect peak(s), this algorithm differentiates the estimated probability density and finds the locations where the signs change from positive to negative. Each peak is determined relatively rather than absolutely because the probability density could be high depending on the bandwidth. Therefore, a peak was defined as the point that fulfills the following two conditions that the peak point exceeds 0.1, and exceeds one quarter of the difference between the maximum value and the minimum values. If any probability density was incomputable due to low occurrence of blinking, the peak-finding algorithm was not applied to those specific results.

We evaluated the kernel-estimated distribution in the range of 0–20 s, which is the usual IBI range. We calculated the median of the results of the simulations for comparison with the means of experimental data, because the shapes of the distributions were diverse. For unimodal distributions, we used these median values to detect the skewness. If the time location of the peak was lower than the centre of the estimated range, we regarded the distribution as the positively skewed.

For bimodal distributions, we evaluated the time locations of two simulated peaks. We permitted differences within ±0.025 s for each reported peak. For instance, if the time locations in the experimental data were 0.5 s and 5.5 s, we assumed that these peaks were reproduced when the first simulated peak was located between 0.475–0.525 s and the second simulated peak was located between 5.475–5.525 s. The width of each histogram bin in Ref. [19] was 0.5 s, and therefore the range was narrow enough to capture the simulated peaks.

## Results

### Distributions of IBI simulated by OSD model

Our simulations resulted in 901, 000 solutions for the OSD model. Then, 70.53%(635, 488/901, 000) of the solutions had a peak, while the remainder (29.46%) had no peak defined by the peakfinder algorithm; bimodal and other multimodal distributions were not detected. One third (30.84%, 195, 985/635, 488) of distributions with a peak were positively skewed although the time location of the peak depended on the parameters. Otherwise (69.15%, 439, 503/635, 488), the simulated distributions approximated normal distributions (See, Fig B in S1 File, for detail). Regarding the distributions without peaks, the probability density was approximately constant within the range of 0–20 s, which is chosen for the simulation. We considered that these results demonstrated peak-less distributions at least in this range. Thus, the one-dimensional stochastic diffusion model reproduced only positively skewed, normal and widespread peak-less distributions of IBIs.

### Proposed model

#### Parameters and behaviours of *V* and distributions of IBI

Contrary to the OSD model, the leaky integrate-and-fire model with a variable threshold reproduced a variety of distributions depending on the parameters. By experimenting with the parameters, we thus could reproduce the distributions of IBI of spontaneous human blinking.

**Fig 2:**
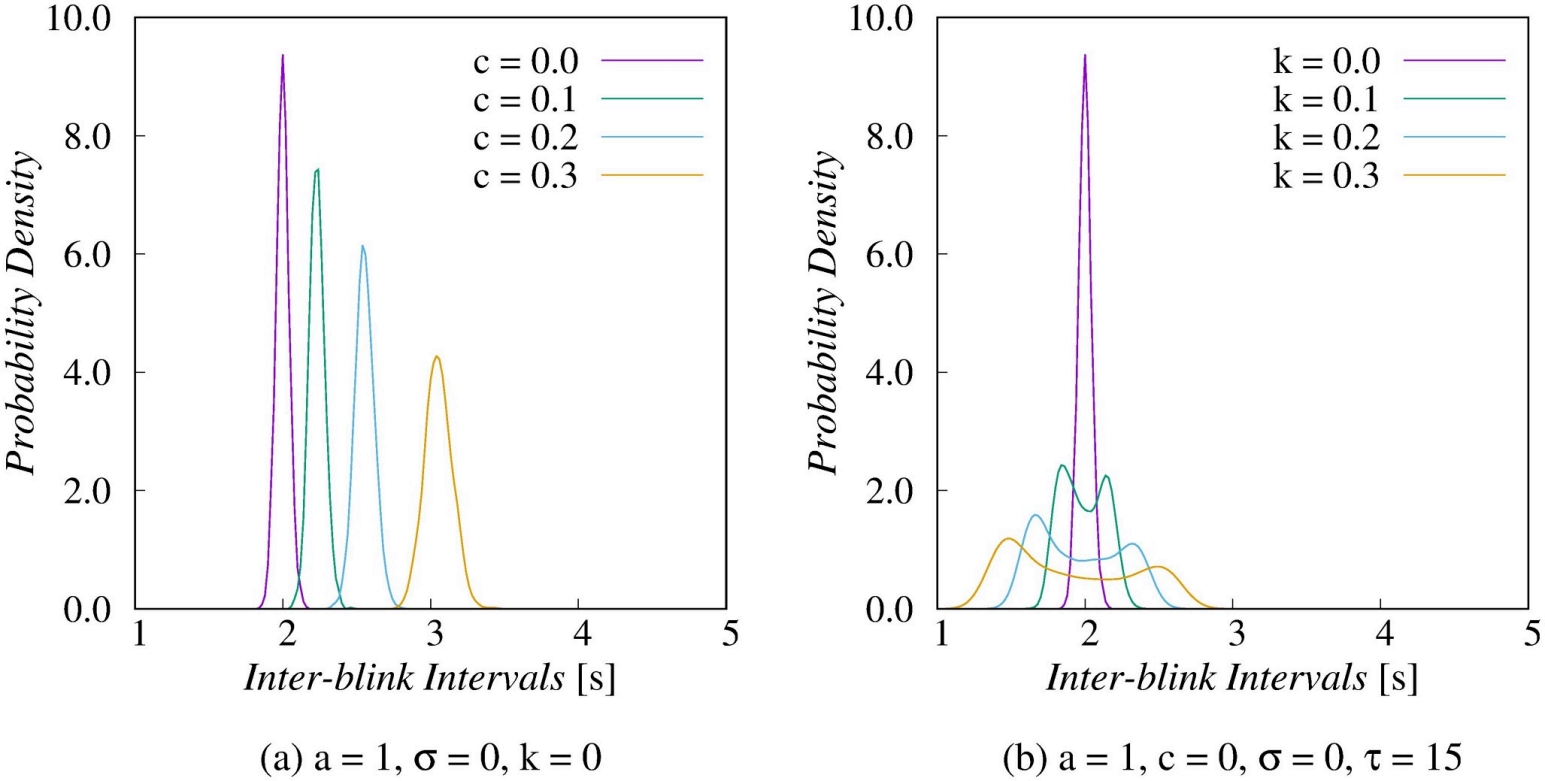
Results obtained by the LIF model with a variable threshold. Probability density functions change in accordance with decay term *c* or amplitude of threshold function *k*. (a) The symmetric shape of distributions are maintained even when the decay term *c* becomes larger. (b) The tails of the distributions expand when the amplitude *k* becomes larger.

When the parameters were fixed at *a* = 1, *σ* = 0, and *k* = 0, the mean and median values increased as *c* became larger within the range of 0.0–0.3 (Fig 2(a)). The symmetric shape of the distribution did not change. In the leaky integrate-and-fire model, the intervals of the external input *I* obey a binomial distribution. Theoretically, the proposed model reproduces the normal distribution of IBI with these specific parameters because a binomial distribution with sufficient sample size approximates a normal distribution.

When the parameters were fixed at *σ* = 0 and *c* = 0 and then the amplitude *k* of the threshold functions varied in the range of 0.0–0.3, the medians of the distributions were almost constant. In this case, however, the tails of the distributions expanded and the shorter IBI showed relatively higher probability density than the longer one (Fig 2(b)).

**Fig 3:**
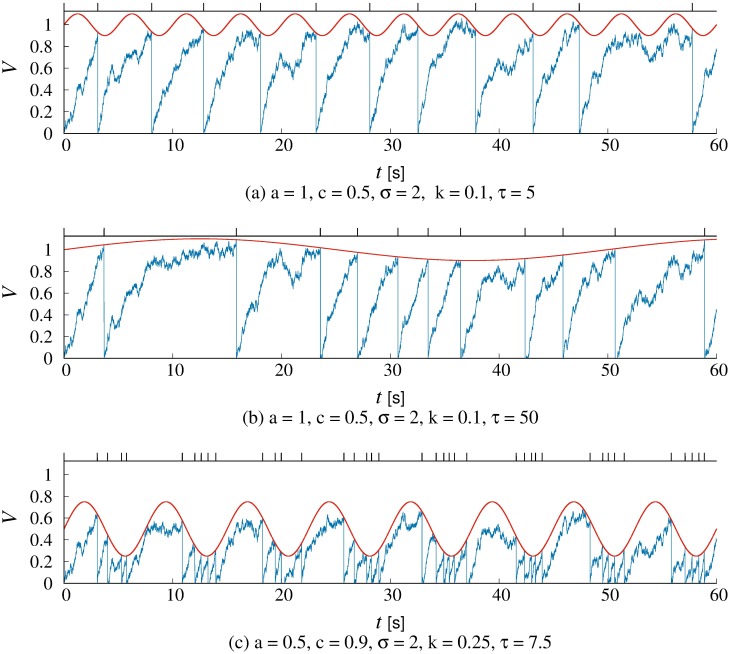
Results by the LIF model with a variable threshold. The *V* increases with integrating the binomial input *I*. The parameter *c* is the decay term and the parameter *σ* is the standard deviation of noise *ξ*. The baseline of the threshold function *a* = 1 and the threshold is time-varing with the amplitude *k* and the period *τ*. (a) The period *τ* is short and the prolonged IBI is observed only if the value *V* is not trapped by the threshold function which is convex down. (b) When the threshold function is convex up with the large period *τ*, the prolonged IBI is frequently observed. (c) Due to the large decay term *c*, the prolonged IBI is observed even when the period *τ* is small.

The proposed model was capable of reproducing bimodal distributions by setting the amplitude *k* and the period *τ* of threshold functions. As shown in Fig 3(a), when the threshold function *θ*(*t*) is convex downward, the value *V* frequently reached the threshold. In this case, the number of the peak was unity. When the threshold function *θ*(*t*) fluctuated near the baseline with a smaller amplitude and a longer period, prolonged IBIs occurred (Fig 3(b)). Due to the effect of the decay term *c*, the value *V* remained just below the threshold. In this case, the number of peaks was two. Therefore, if a larger decay term was chosen, we were able to obtain both relatively longer IBIs and shorter IBIs even when the baseline was much lower (Fig 3(c)).

We chose the parameters of the proposed model as shown in Table 2 to cover approximately widest ranges of *c* and *k*. The third column in Table 2 shows increments for the parameters *c*, *k*, and *τ* (See, S2 File for a sample of the results). The period 1 ≤ *τ* ≤ 10 s was set to correspond to the range 0.1–1.0 Hz. For the sake of simplicity, other parameters were fixed to *a* = 1 and *σ* = 0.

**Table 2:** Parameters used in experiments by the LIF model with a variable threshold.

	range	an increment
*c*	[0, 1]	0.01
*k*	[0, 0.9]	0.01
*τ*	[0.5, 10]	0.5

In the range of these parameters, we obtained 174,629 solutions for the proposed model. The results of peak-detection showed that 4.68% (8, 170/174, 629) of distributions were peak-less, 37.95% (66, 273/174, 629) were unimodal, 41.03% (71, 653/174, 629) were bimodal, and 1.38% (2, 411/174, 629) of those were trimodal. The remaining 14.96% (26, 122/174, 629) of distributions were not computable due to their lower number of blinks.

**Fig 4:**
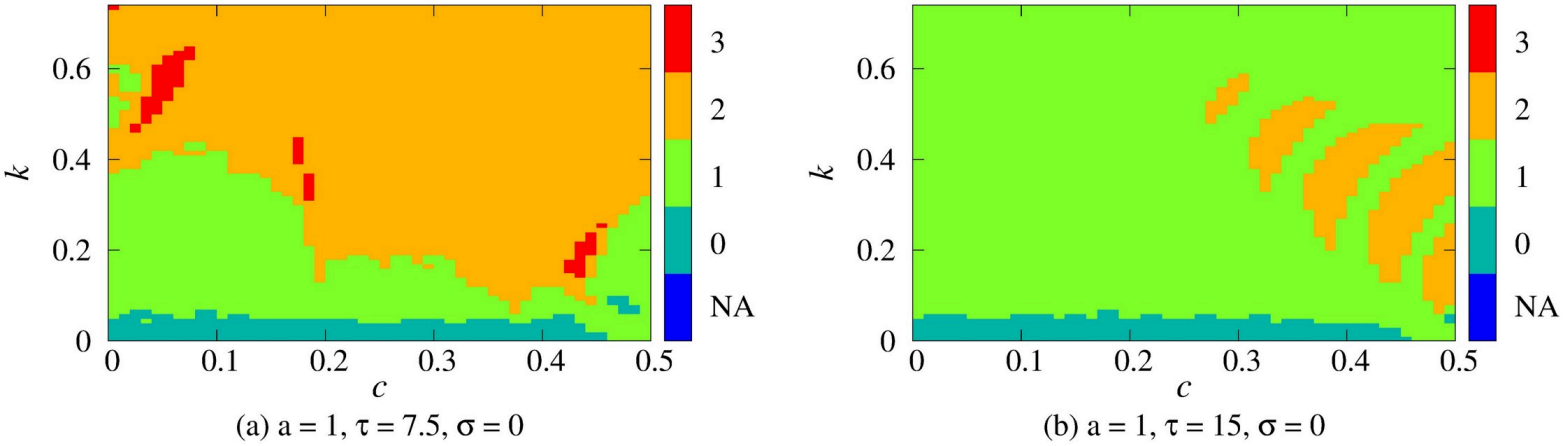
The number of peaks of the distributions of IBI in case that *c* and *k* are changed. The color bars show the number of peaks. (a) Trimodal distributions are observed as red clusters surrounded by the areas of bimodal distributions. (b) For the larger period *τ*, trimodal distributions are not observed.

The proposed model also produced trimodal distributions. Fig 4 demonstrates the number of peaks depending on decay term *c* and amplitude *k* when *a* = 1 and *σ* = 0 (these parameters are discussed in the next section).

#### Reproduction of Ponder and Kennedy’s [19] bimodal distributions of IBI

The proposed model is capable of reproducing bimodal distributions of IBIs. In this reproduction, the time bins that contain peaks were determined by the combination of baseline *a* and amplitude *k* of the threshold function *θ*(*t*). The value *V* is most likely to reach the threshold when the threshold function *θ*(*t*) has a minimal value at

$$\sin\frac{2\pi t}{\tau }=-1,$$

where

$$\theta \left(t\right)=a+k\sin\frac{2\pi t}{\tau }=a-k.$$

Hence, the time location of the first peaks (the peak closest to 0) is determined by the values *a* − *k*. If the decay term exists in the range of 0 < *c* < 1, the first peak is located around 0.5 s when *a* − *k* ≃ 0.15. If the value *V* is not trapped by the threshold function, it increases with non-negative inputs. Then, the value *V* certainly hits the threshold function which is convex downward. Therefore, the intervals between the time location of the first peak and that of the second peak are always smaller than the period *τ* of the threshold function. Consequently, the time location of the second peak depends on the period *τ*.

Assuming that the threshold function determines time locations of peaks, we can reproduce two peaks where we intend to allocate. Table 3 demonstrates the time locations of peaks and the means in the bimodal distributions in the experimental study [19].

**Table 3:** Peaks and means reported in Ref. [19] and the parameter ranges to reproduce these peaks.

Reported in Ref. [19]				Parameters of the proposed model			
Case	First peak	Second peak	Mean	*a* − *k*	*τ*	Freq.[Hz]	Median
1	0.5	3.5	2.05	0.14–0.19	4.0–7.0	0.14–0.25	2.42–2.73
2	0.5	5.0	3.31	0.14–0.16	6.0–8.5	0.12–0.16	3.45–3.86
3	0.5	5.0	3.64	0.14–0.16	6.0–8.5	0.11–0.16	3.45–3.86
4	0.5	6.5	4.12	0.15–0.16	8.0–8.5	0.11–0.13	4.65–4.91
5	1.0	5.5	3.95	0.30–0.35	6.5–9.0	0.11–0.15	3.95–4.65
6	0.5	7.0	4.45	0.15	9.0	0.11	5.03

For case 6, one combination of parameters existed.

The parameters shown in Table 3 demonstrate the minimum value *a* − *k* and the period *τ* that reproduce bimodal distributions. As shown in Table 3, 0.14 ≤ *a* − *k* ≤ 0.35 and the period was 4.0 ≤ *τ* ≤ 9.0 s. These periods correspond to 0.11–0.25 Hz.

Furthermore, the proposed model also produces trimodal distributions if particular parameters are given. For instance, we obtain trimodal distributions when *c* = 0.05, *a* = 1, and *k* = 0.6, i.e., *a* − *k* = 0.4 under the condition that the period *τ* = 7.5. The combinations of parameters that reproduce trimodal distributions were distributed as clusters (red regions in Fig 4(a)). The trimodal distributions were also obtained when we expanded the ranges of parameters to 0 ≤ *c* ≤ 1 and 0 ≤ *k* ≤ 0.9 (See, Fig C in S1 File). The trimodal distributions could exist in areas surrounded by the bimodal distributions (Figs C (b) and C (c) in S1 File). To reproduce the empirical bimodal distributions reported by Ponder and Kennedy [19], the parameter range of *τ* was estimated as 4.0–9.0. Within this range, we obtain the trimodal distributions as well (Figs C (b) and C (c) in S1 File).

## Discussion

### Distributions of spontaneous human blinking

Although the OSD model [9] reproduced the positively skewed, normal, and peak-less distributions of spontaneous human blinking, the model did not reproduce bimodal distributions within the range of typical parameters. In contrast, the proposed model reproduced all four distributions including the bimodal one.

Contrary to the previous experimental study [19], the positively skewed distribution was not the most common among the numerical results of the proposed model: 66, 273 cases (37.95%) followed a unimodal distributions and only 22, 142 (12.6%) cases were positively skewed. The normal distributions were also achieved by the binomial nature of inputs, albeit only in the simplest cases with noiseless inputs and thresholds with a constant value, i.e. *σ* = 0 and *k* = 0. In most simulations, however, *σ* = 0 and *k* > 0. These results suggest that a noisy system reproduces the positively skewed distributions if the threshold varies periodically. One possibility is that positively skewed distributions are common in previous studies (e.g., [3], [19]) as a consequence of the ubiquitous noise in biological systems, such as blink generators.

The bimodal distribution was also observed in the experimental study [19], albeit less commonly than the positively skewed and normal distributions. To reproduce the bimodal distributions, the differences between baseline and threshold amplitude, i.e. *a* − *k*, had to be set at lower values. When the value of the threshold function was convex downward (Fig 3(c)), the model elicited a series of blinks within short intervals. Frequent blinking in a short period, known as “blink bursts” [9], could be explained by the short term decrease of the threshold function.

In this paper, the proposed model also produced trimodal distributions. The combinations of the parameters that produce the trimodal distributions were not localized but distributed in small regions (Fig 4). In future research, we will examine whether or not trimodal distributions of IBI can be confirmed experimentally. As one of the cases, we consider a viewing task that requires visual attention. In such a simple perceptional task, we could assume that cognitive load, i.e., *I*, is almost task-independent, or obey a stochastic process. The saliency and the stimulus value is well controlled and thus the visual attention is simply regulated by the presentations of visual targets. Here, *k* and *c* could be interpreted as individual factors, sensitivity to the external stimuli and tendency to induce blink suppressions, respectively. When sensitivity of a participant becomes higher, this is represented as a larger value of *k* in the model. The parameter *c* is a decay term and thus if *c* is larger, the value *V* tends to fluctuate under the threshold, producing prolonged IBIs. Therefore, a larger *c* corresponds to the tendency to induce blink suppressions.

Trimodal distributions might be observed when we change the conditional variables that correspond to *k* and *c* in experiments with participants who show bimodal distributions. First, the targets of visual attention is intermittently presented within 7.5 s, which corresponds to *τ*. Second, when sensitivity of a participant *k* is relatively low, e.g., *k* = 0.2, the shortest IBI would be averagely 1.6 s when there is no decay *c* = 0. Meanwhile, a participant has a moderate tendency of blink suppression, in the range of *c* = 0.41–0.45, trimodal distributions could be observed. For this participant, the value *V* fluctuates under the threshold function because decay and the input intensity are well balanced, producing prolonged IBIs. However, once the threshold is convex downward due to disappearance of targets, the value *V* must hit the threshold function in several hundred milliseconds, resulting a termination of the prolonged IBI. Two cases can occur after the reset. In one case, it takes a few seconds until the *V* reaches to the threshold again because the previous reset occurred approximately at the maximum value of the threshold function. In another case, short-term sequential blinking is observed if the previous reset occurred at near the minimum value of the threshold function. As the results, prolonged IBI and two types of behaviours after reset would produce the trimodal distributions of IBI.

In more complex task, *k* corresponds to the integration of task-dependent cognitive loads as well as individual sensitivity to the external stimuli. Thus, we need considerations on certain characteristics of the variable threshold when we argue more complex tasks by applying the proposed model.

### The variable threshold and biological oscillations

The results of numerical simulations in this study suggest that the variable threshold plays a critical role in producing a variety of IBI distributions, especially for the bimodal distribution. Numerous experimental studies have revealed that the blink rates are regulated by internal states of the participants during performing cognitive tasks (e.g., [6, 11]). While we assumed that the variable threshold represented particular physiological fluctuations, a few plausible candidates of human internal states exist.

Researchers have reported that dopamine levels in the brain may influence IBI. For example, pathologic reduction of dopamine induces a lower frequency of blinking and fewer variations of IBI [3]. The blinking rate varies depending on the level of tonic and phasic dopamine [22]. In other words, the frequency of blinking varies in accordance with the innate baseline and transient states of the dopamine levels. As one possibility, one could speculate that the threshold fluctuations in the proposed model correspond to phasic dopamine levels. If this hypothesis is correct, blinking frequencies increase with phasic dopamine levels, reshaping the distributions of IBI.

Rhythms of human biological systems such as brain waves [23] and attentional fluctuations [24] could also be candidates. The results of reproduction of the bimodal distributions suggested that relatively slow oscillations (0.11–0.25 Hz) regulate blinks. Recent neurological studies have found delta-band (0.5–4 Hz) blink-related oscillations (BROs) in a resting sate [25]. One study [23] reported that spontaneous blinks activate precuneus regions related to awareness and monitoring of the environment. Physiological fluctuations represented by the threshold function in the proposed model may relate to such brain waves.

### Consistency between the model and the physiological foundations of motor control

In the proposed LIF model, *V* represents the changes in an internal value of a blink generator. Although the location of the blink generator circuit is controversial [3], human blinking must be involved in the general motor control circuits. There is no major contradiction if we assume that the integration of cognitive load may correspond to a direct path of excitatory motor control circuits that increase blinking frequency. On the other hand, inhibitory signals decrease blinking frequency and therefore can provide less frequent blinks, leading variations of IBI [2], [3]. The variations of the threshold would be in accordance with an indirect path of inhibitory motor control circuits. The results on IBI distributions in this paper suggest that a variable threshold can create two or three types of IBI. When we acknowledge the variable threshold in the LIF model corresponds to this inhibitory control, we can argue that human blinking rates vary in a few tens of seconds due to the effect of inhibitory signals [5]. While the LIF models are often used for a neuron, it also seems that the model would be useful to represent human blinking as the macroscopic phenomenon that involves multiple brain areas.

## Conclusion

In this paper, we proposed a leaky integrate-and-fire model with a variable threshold to model human spontaneous blinking. The proposed model could reproduce the positively skewed, normal, and peak-less distributions of IBI. Moreover, the proposed model reproduced the bimodal distributions, which could not be reproduced by the OSD model at least within the typical range of parameters.

Parameters that reproduce the temporal locations of peaks in the experimental distributions reported by a classical study [19] suggest that relatively slow oscillations (0.11–0.25 Hz) govern blink elicitations. The proposed model also predicts the existence of the trimodal distributions of IBI and the distributions could be produced by the non-specific parameters. As a possible mechanism, we can assume that changes in blink rates would reflect fluctuations of threshold regulated by particular human internal states such as a brain dopamine level or rhythms of human biological system.

## Supporting information

S1 File Replications of one-dimensional-stochastic diffusion model, Figs A-C, Table A, and information of the used program. (PDF)

S2 File A sample of the results calculated using the proposed model. (TXT)
