# Supplementary material for: Threshold-varying integrate-and-fire model reproduces distributions of spontaneous blink intervals
Source: PLoS One. 2018 Oct 30;13(10):e0206528. doi: 10.1371/journal.pone.0206528 (PMC6207319; doi:10.1371/journal.pone.0206528)
Supplement: S1 File — (PDF) [file pone.0206528.s001.pdf]

S1 File

Threshold-Varying Integrate-and-Fire Model Reproduces Distributions of Spontaneous Blink Intervals

Ryota Nomura, Ying-Zong Liang, Kenji Morita, Kantaro Fujiwara, Tohru Ikeguchi

This file includes:
Replications of one-dimensional stochastic diffusion model
Figures A-C
Table A
Information of the used program

Replications of one-dimensional stochastic diffusion model

Model and conditions of simulations

The one-dimensional stochastic diffusion (OSD) model [1] has been proposed as a mathematical model of spontaneous blinking. In this model, changes in the potential X of the blink generator are governed by the following equation:

$$dX(t) = \left(-\frac{X(t)}{\beta} + \mu\right) dt + \phi dW(t), \qquad \text{S(1)}$$

with an initial condition $X(0) = X_0$. In Eq. S(1), W is a Wiener process that is characterized by spontaneous decay β (> 0), average input μ ($-\infty < \mu < \infty$), and a noise term of ϕ (> 0) for a random process. In this model, intervals between blinks are formulated as a first-passage-time to a constant threshold.

Table A. Parameters used in the OSD model. Values are increased by the corresponding values in the third column.

	range	an increment
β	[0.01, 10.0]	0.01
μ	[0.1, 10.0]	0.1
ϕ	[0.5, 1.0]	0.05

Here, we reexamined the reproducibility of the distributions of inter-blink intervals (IBIs) by the OSD model. In this replication, the threshold potential was set to 1.0 and the parameters were set as shown in Table A to cover the typical ranges of decay β and input μ that elicit blinking at realistic intervals. Regarding the distributions without peaks, the probability density was approximately constant within the range of 0–20 s, which is chosen for the numerical experiments. We considered that these results demonstrated peak-less distributions at least in this range.

Potential behaviour and distributions of IBI simulated by the OSD model

We used the peakfinder algorithm [2] to detect the number of peak(s). Our simulations resulted in $901,000$ solutions for the OSD model. Then, 70.53% ($635,488/901,000$) of the solutions had a peak, while the remainder (29.46%) had no peak. No bimodal and other multimodal distributions were detected.

One third (30.84%, $195,985/635,488$) of distributions with a peak were positively skewed although the time location of the peak depended on the parameters. Otherwise (69.15%, $439,503/635,488$), the simulated distributions approximated normal distributions.

Figs B (a)–(c) shows the numbers of peaks depending on the parameters β and μ. When ϕ increased, the ratio of the distributions with a peak also increased. However, the bimodal distributions were not reproduced by these combinations of the parameters. Namely, the OSD model could not reproduce the bimodal distributions at least within the typical range of the parameters.

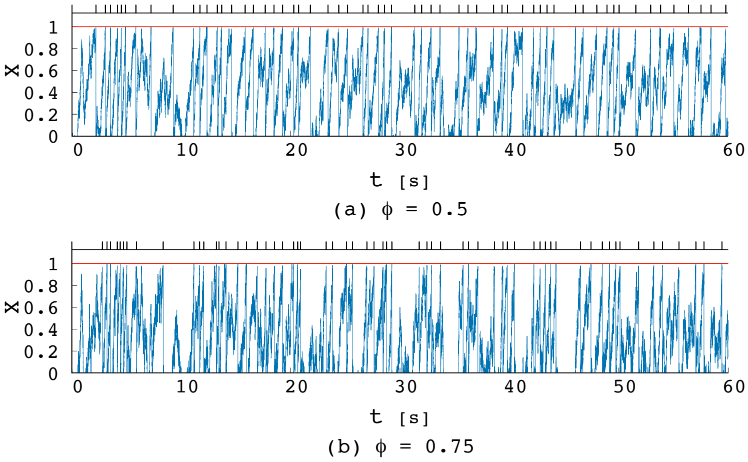

Figure A. Results of numerical experiments by the OSD model. The X increases with integrating the input. The parameter β is the spontaneous decay, the parameter μ is the average input, and the ϕ is the noise term for a random process. $\beta = 10.0$ and $\mu = 1.0$.

The number of peaks of IBI distributions simulated by the proposed model

As we have shown in the main text, the proposed model reproduced the peak-less, unimodal, and bimodal distributions (Fig 4(a) in the main text and Fig C). Furthermore, the proposed model also produced the trimodal distributions when particular combinations of parameters were set.

When we expanded the ranges of parameters to $0 \leq c \leq 1$ and $0 \leq k \leq 0.9$, the trimodal distributions were obtained in wider areas (red regions in Fig C(b)–(c)). To reproduce the empirical bimodal distributions reported in Ref. [3], the parameter range of τ was estimated as 4.0–9.0. Within the range of τ, the trimodal distributions could exist in areas surrounded by the bimodal distributions as shown in Fig C (b)–(c).

Information of the used program

The used program of the proposed variable-threshold LIF model was deposited in BioModels Database [4] and assigned the identifier MODEL1810190001. We also provide an example of the results simulated using the proposed model in S2 file. S2 file includes calculated values of time, V, a threshold function, and blink flag (each one shows a blink) of the first $10,000$ steps, from 0.000 s to 9.999 s.

References

1. Hoshino K. Ornstein-Uhlenbeck first-passage-time models for spontaneous eye blinking. Proc 18th Annu International Conference of the IEEE 1996; 5: 1784–1785. doi: 10.1109/IEMBS.1996.646254

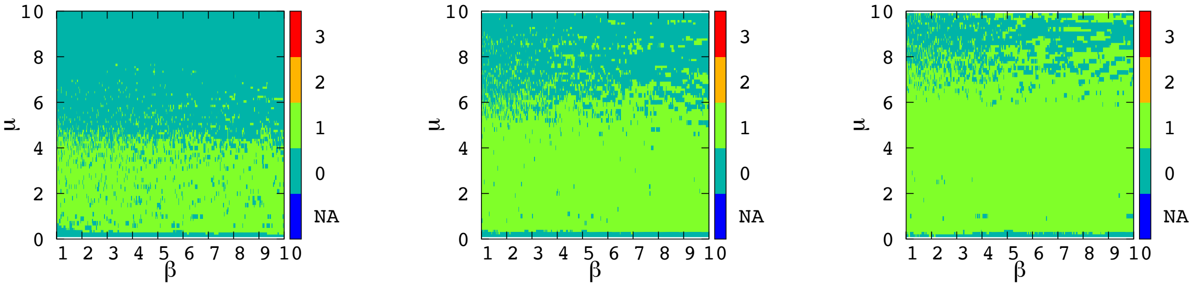

(a) ϕ = 0.5 (b) ϕ = 0.75 (c) ϕ = 0.95

Figure B. Results of the OSD model. The number of peaks depends on β and μ. The X increases with integrating the input. The parameter β is the spontaneous decay, the parameter μ is the average input, and the ϕ is the noise term for a random process. Color bars show the number of peaks. NA in the color bar indicates that no results were applicable due to low occurrence of blinking.

2. Yoder N. Peakfinder. 2011; Internet: http://www.mathworks.com/matlabcentral/fileexchange/25500

3. Ponder E, Kennedy W. On the act of blinking. Exp Physiol. 1927; 18(2):89–110.

4. Chelliah V. et al. BioModels: ten-year anniversary. Nucl Acids Res 2015; 43(Database issue):D542-8.

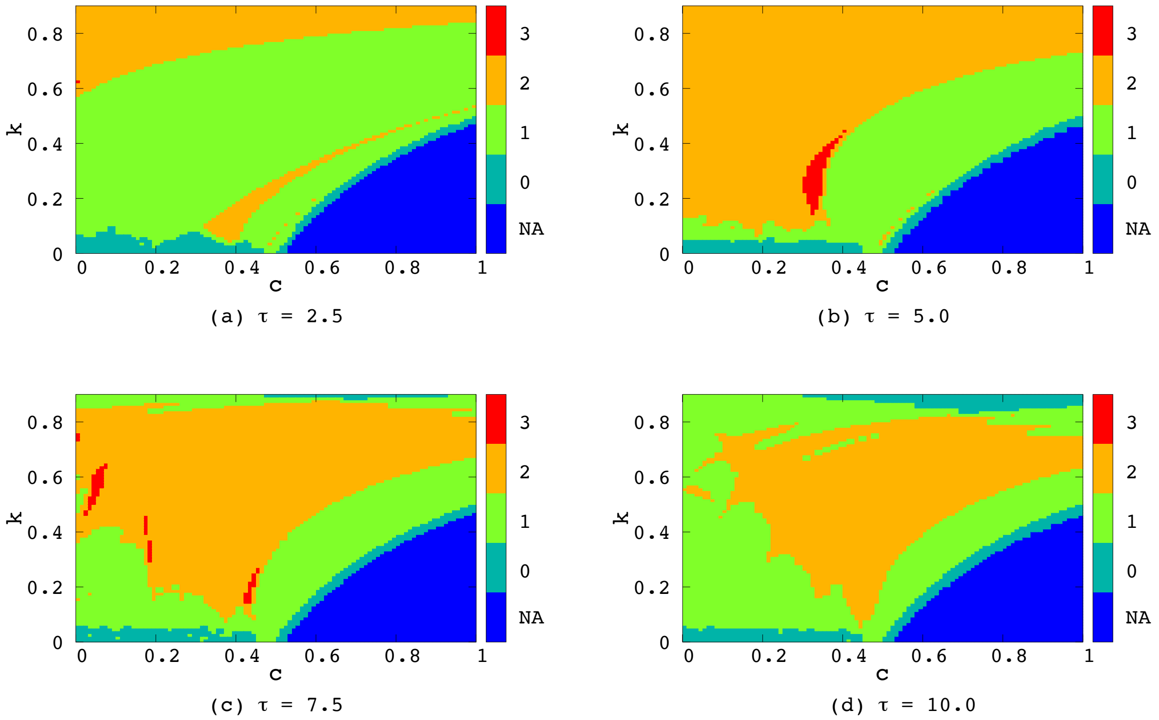

Figure C. Results of the proposed model. The number of peaks depends on c and k. (a)–(d) $a = 1$ and $\xi = 0$. The parameter c is the decay term. Regarding the threshold function, the parameter k is the amplitude and the parameter τ is the period. Color bars show the number of peaks. NA in the color bars indicates that no results were applicable due to low occurrence of blinking. Fig 4(a) in the main text is an enlargement of Fig C(c) with the parameters $0 \leq c \leq 0.5$ and $0 \leq k \leq 0.75$.
